# Effect of Mn(II) and Co(II) on Anti-*Candida* Metabolite Production by *Aspergillus* sp. an Endophyte Isolated from *Dizygostemon riparius* (Plantaginaceae)

**DOI:** 10.3390/ph17121678

**Published:** 2024-12-12

**Authors:** Anne Karoline Maiorana Santos, Bianca Araújo dos Santos, Josivan Regis Farias, Sebastião Vieira de Morais, Cleydlenne Costa Vasconcelos, Rosane Nassar Meireles Guerra, Edson Rodrigues-Filho, Alberto Jorge Oliveira Lopes, Antônio José Cantanhede Filho

**Affiliations:** 1 Chemistry Postgraduate Program, Federal Institute of Science Education and Technology of Maranhão, São Luís 65030-005, Brazil; annemaiorana10@gmail.com (A.K.M.S.); biancaaraujo57@hotmail.com (B.A.d.S.); svmcoluna@gmail.com (S.V.d.M.);; 2 Center for Biological and Health Sciences, Federal University of Maranhão, São Luís 65080-805, Brazil; 3 LaBioMMi, Federal University of São Carlos, CP 676, São Carlos 13565-905, Brazil

**Keywords:** anti-*Candida* metabolites, endophytic fungi, metal-induced secondary metabolism, natural products biotechnology

## Abstract

**Background/Objectives**: This study evaluates the effect of Mn(II) and Co(II) ions on the production of anti-*Candida* metabolites by the endophytic fungus Aspergillus sp., isolated from *Dizygostemon riparius*. The objective was to identify metal-induced secondary metabolites with antifungal potential against drug-resistant *Candida* species. **Methods**: *Aspergillus* sp. was cultivated in Czapek agar supplemented with MnCl₂ (400 µM) or CoCl₂ (200 µM). Metabolite profiles were analyzed using UHPLC-DAD and LC-ESI-HRMS, followed by structural elucidation via NMR. Antifungal and biofilm inhibition activities were tested against *Candida albicans* and *Candida parapsilosis*. Toxicity was assessed using *Tenebrio molitor* larvae. **Results**: Key metabolites, including pyrophen, penicillquei B, and fonsecinone B, demonstrated antifungal activity with MIC values of 4.37–280.61 µg/mL. Fonsecinone B exhibited superior biofilm inhibition, surpassing fluconazole in reducing biofilm biomass and viability. In vivo assays showed low toxicity, with survival rates above 80% at 2× MIC/kg. **Conclusions**: Mn(II) and Co(II) significantly modulated the production of antifungal metabolites in *Aspergillus* sp. Fonsecinone B emerged as a promising candidate for antifungal therapy due to its potent activity and low toxicity. These findings support further investigation into the therapeutic potential of metal-induced fungal metabolites for combating drug-resistant *Candida* infections.

## 1. Introduction

Fungi are renowned for their extensive production of bioactive molecules, particularly secondary metabolites, crucial for new drug discovery [1,2]. These metabolites, constituting over two-thirds of antibiotics and half of anticancer drugs currently in clinical use, underscore the biotechnological potential of fungi [3].

Fungi produce 42% of the 23,000 bioactive microbial products identified. Among the fungi, Ascomycetes such as *Aspergillus*, *Penicillium*, and *Fusarium* are notable, producing approximately 950, 900, and 350 metabolites, respectively. These fungal metabolites can be functionally distinct, including biologically active compounds that are either harmful (e.g., toxins) or beneficial (e.g., antimicrobials) to humans [4].

Nonetheless, with the addition of metal cations in culture media, which apparently can modulate enzyme activation and gene expression linked to metabolite production, this prolific bioactive metabolite production can even be enhanced. Although not yet well rationalized, metal cations such as Mg(II), Mn(II), Fe(II), and Zn(II) added to culture media have the potential to significantly impact fungal metabolism by acting as enzyme cofactors and altering redox potential, influencing both the quantity and diversity of secondary metabolites produced [5,6].

Endophytic fungi, residing mutualistically within plant tissues, are emerging as vital sources for new bioactive compounds, especially in stress conditions that can lead to enhanced secondary metabolite production [7,8,9]. *Aspergillus*, a significant fungal genus, produces various secondary metabolites including terpenes and polyketides with strong antimicrobial properties by inhibiting essential microbial processes such as cell wall and DNA synthesis [10,11,12].

In this context, the development of candidate drugs against infections, especially those caused by fungi, is essential, given that the resistance profile of fungi is alarming for public health and generates high costs for treatments [13]. Among the most reported species, *Candida albicans* and *C. parapsilosis* are classified as critical-risk pathogens by the World Health Organization (WHO), as they present different mechanisms of invasion and cause severe infections [14,15,16,17,18]. Therefore, the aim of this study was to investigate the efficacy of metabolites from endophytic fungi isolated from *Dizygostemon riparius* against *Candida* species, aiming to contribute to the development of new antifungal therapies, with metal cations playing a crucial role in optimizing the production of these bioactive compounds in culture media.

## 2. Results

### 2.1. Preparative-Scale Cultivation of the Endophytic Fungus Aspergillus sp. and Extraction

In preparing the broths for both cultivations, the Czapek liquid medium was used as a control, labeled “C”. For the second and third cultivations, FeSO_4_ was omitted, and the media were supplemented with halogenated salts, MnCl_2_ and CoCl_2_, each at a concentration of 25.0 mmol/mL, labeled “Mn” and “Co”, respectively. After sterilization, the media were inoculated with the endophyte and incubated for 21 days at room temperature in static mode.

At the end of fermentation, the mycelium was separated by filtration and the fermentation broth was partitioned in duplicate with EtOAc (1:1, *v*/*v*). The organic solvent was then evaporated under reduced pressure to generate the EtOAc phase from the three fungal cultivation conditions. The mycelium was further extracted with MeOH (150 mL) to produce the methanolic crude extract for the three cultivations.

### 2.2. UHPLC-DAD Analysis of EtOAc and EtOH Fractions

The chromatographic profiles of medium- to high-polarity fungal metabolites were compared due to their complexity across experiments involving Mn(II), Co(II), and the control, using the UHPLC-DAD technique. This approach facilitated the analysis and differentiation of metabolites induced at an early stage. The analyzed fractions were derived from the EtOAc phase obtained from the liquid medium in which the fungus was cultivated and from the EtOH extract of the mycelium, respectively. Induced substances were observed starting from fraction 4. In the comparative chromatograms obtained via UHPLC-DAD, the organic phase from the liquid medium with manganese (MnAF4) (retention time (RT): 6.15, 9.76, and 9.37 min) and the organic phase from the liquid medium with cobalt (CoAF4) (RT: 10.20 and 11.06 min) displayed differences from the control of the EtOAc phase (CAF4). Furthermore, more pronounced inductions were observed when compared with the control of the EtOH extract of the mycelium (BCAMF4) in the EtOH extract of the mycelium cultivated with manganese (BMnAMF4) (RT = 5.95, 6.54, 8.70, 9.74, 9.91, and 10.25 min) and in the EtOH extract of the mycelium cultivated with cobalt (BCoAMF4) (RT = 6.33, 6.91, 8.11, 9.77, 9.93, and 10.27 min) (Figure S1A).

In fraction 5, induced substances can also be observed when compared to the control of the EtOAc phase (CAF5) in the culture with (Mn II-MnAF5), with RT of 5.73, 5.98, and 6.20 min. Similarly, in the presence of (Co II-CoAF5), the substances with RT of 9.37, 9.75, 9.92, and 10.28 min are examples of induced compounds. In the EtOH extract, the culture with the use of cations exhibits a similar chemical profile, yet differences are also present when compared to the control of the EtOH extract (BCAMF5). The induced substances with RT of 6.90, 7.36, 7.80, 8.14, 8.78, and 9.17 min are examples of these differences (Figure S1B).

The comparative chromatograms obtained by UHPLC-DAD from fraction 6 similarly to those presented earlier, revealed induced substances. In the culture with manganese (MnAF6), metabolites with RT of 4.01, 5.58, 5.74, and 5.99 min were observed. For cobalt (CoAF6), the compounds with RT of 5.79, 7.62, 8.27, 8.58, and 9.36 min are examples of induced compounds when compared to the control of the EtOAc phase (CAF6). In the EtOH extract, similar observations can be made; for manganese (BMnMF6), RTs of 5.49, 6.48, 7.34, 7.81, 8.12, 8.61, and 9.14 min were noted, as well as for cobalt (BCoMF6), with RT of 5.50, 6.48, 8.62, and 9.16 min, when compared to the control (BCMF6) (Figure S1C).

### 2.3. Annotation of Compounds Present in the EtOAc Phases and EtOH Extracts

**Figure 1:**
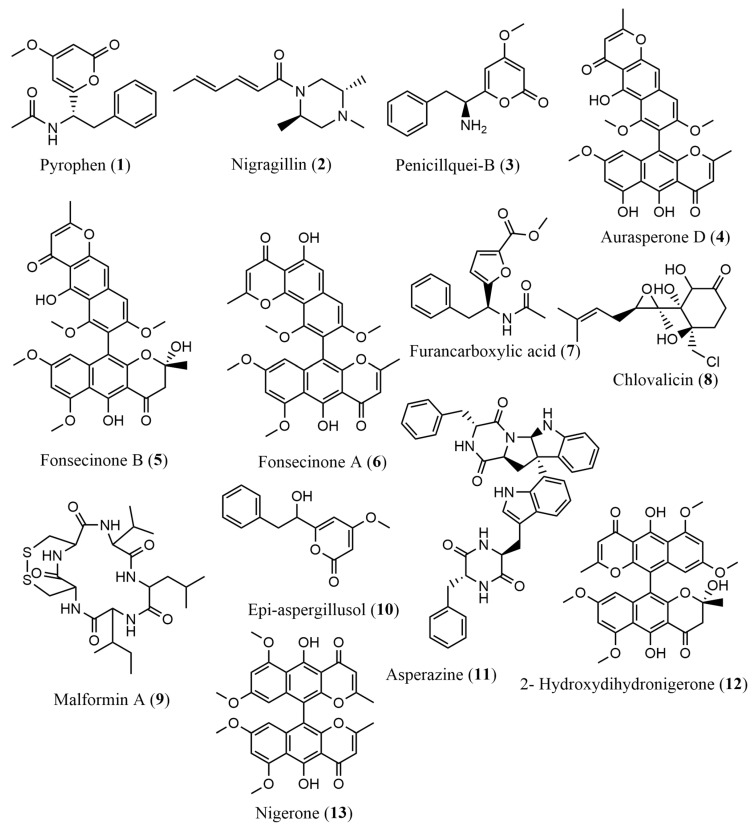
Compounds annotated by LC-ESI-HRMS in positive mode.

Based on the analyses obtained through LC-ESI-HRMS in positive mode, thirteen compounds were annotated from both the EtOAc phases (CA, MnA, and CoA) and the EtOH extracts (CM, MnM, and CoM), as described in Table 1 and illustrated in Figure 1. Compounds **1**, **2**, and **3** were annotated in the EtOAc phases as pyrophen (**1**), *m*/*z* 288.1169 ([M+H]^+^, calc. 288.1230 for C_16_H_18_NO_4_^+^, ∆ = 0.35 ppm); nigragillin (**2**), *m*/*z* 223.1804 ([M+H]^+^, calc. 223.1805 for C_13_H_23_N_2_O^+^, ∆ = 0.44 ppm); and penicillquei B (**3**), *m*/*z* 246.1122 ([M+H]^+^, calc. 246.1125 for C_14_H_16_NO_3_^+^, ∆ = 1.22 ppm).

Compounds **4**, **5**, and **6** were annotated in the EtOH extracts, identified as belonging to the naphthopyrone family: aurasperone D (**4**), *m*/*z* 557.1436 ([M+H]^+^, calc. 557.1442 for C_31_H_25_O_10_^+^, ∆ = 1.07 ppm); fonscecinone B (**5**), *m*/*z* 589.1705 ([M+H]^+^, calc. 589.1704 for C_32_H_29_O_11_^+^, ∆ = 0.17 ppm); and fonscecinone A (**6**), *m*/*z* 571.1597 ([M+H]^+^, calc. 571.1599 for C_32_H_27_O_10_^+^, ∆ = 0.35 ppm).

Additionally, six more compounds were identified through LC-ESI-HRMS in positive mode as furancarboxylic acid (**7**) observed in the EtOAc phases CA and CoA, with *m*/*z* 288.1229 ([M+H]^+^, calc. 288.1230 for C_16_H_18_NO_4_^+^, ∆ = 0.35 ppm); chlovalicina (**8**), with *m*/*z* 332.1390 ([M+H]^+^, calc. 332.1391 for C_16_H_26_ClO_5_^+^, ∆ = 0.3 ppm); malformin A1 (**9**) was observed in the EtOAc phases, with *m*/*z* 530.2463 ([M+H]^+^, calc. 530.2465 for C_23_H_40_N_5_O_5_S_2_^+^, ∆ = 0.37 ppm); epi-aspergillusol (**10**) identified in the EtOAc phases CA and MnA, with *m*/*z* 247.0977 ([M+H]^+^, calc. 247.0965 for C_14_H_15_O_4_^+^, ∆ = 4.85 ppm); asperazine (**11**), present in the EtOH extract and MnM, with *m*/*z* 665.2871 ([M+H]^+^, calc. 665.2863 for C_40_H_37_N_6_O_4_^+^, ∆ = 1.20 ppm); 2-hydroxydihydronigerone (**12**) identified in the EtOH extracts MnM and CoM, with *m*/*z* 589.1716 ([M+H]^+^, calc. 589.1704 for C_32_H_29_O_11_^+^, ∆ = 2.03 ppm); and nigerone (**13**), with *m*/*z* 571.1600 ([M+H]^+^, calc. 571.1599 for C_32_H_27_O_10_^+^, ∆ = 0.17 ppm).

### 2.4. Analysis by LC-ESI-HRMS of the EtOAc Extracts

**Figure 2:**
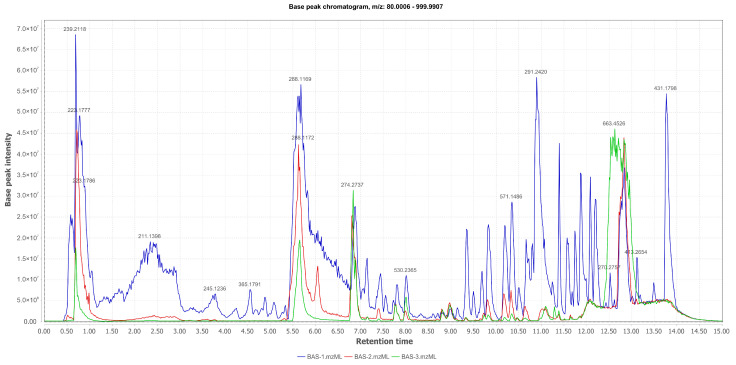
Comparative chromatogram of total ions of crude extracts of the EtOAc phase (EtOAc Control in blue; Mn_EtOAc in red; Co_EtOAc in green) derived from the liquid culture medium of *Aspergillus* sp.

The chemical profile observed from the chromatograms obtained by LC-ESI-HRMS in positive mode of the crude EtOAc extracts from the liquid medium of *Aspergillus* sp. culture (Figure 2) clearly demonstrates a significant alteration caused by the presence of Mn(II) and Co(II) cations in the medium. The fungus was able to produce a range of secondary metabolites in the liquid Czapek medium used as the control. However, in the experiment this production is drastically reduced with the use of MnCl_2_ and CoCl_2_. In the culture with Co(II), the fungus still managed to produce two substances ([M+H]^+^ at *m*/*z* 274.2730 and 663.4542) at higher abundance than in the control. Therefore, it can be concluded that the fungus inhibited metabolite synthesis when comparing the total ion chromatograms of the control culture medium with those of the media supplemented with Mn(II) and Co(II). This result may be related to the concentration of analytes used in the fermentation medium, the toxicity of the metals to the fungus, or the complexity of the extracts in the context of the experiment.

### 2.5. Comparison of Chemical Profiles by LC-ESI-HRMS of Fractions Obtained from Czapek and Mn(II)- and Co(II)-Supplemented Media

**Figure 3:**
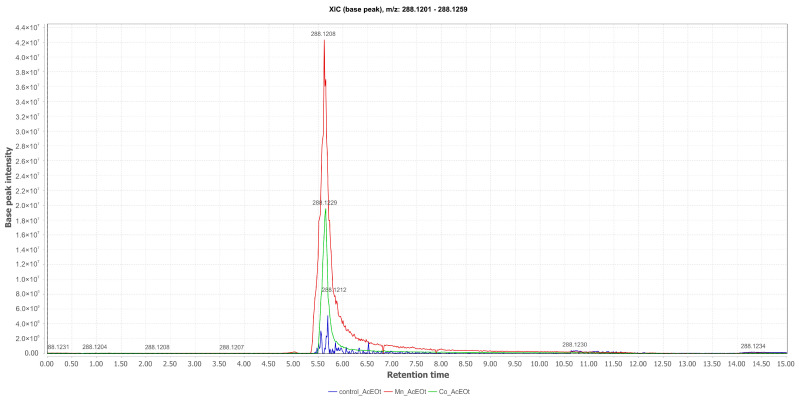
Comparative selected ion chromatograms for pyrophen with *m*/*z* 288.1229 (EtOAc Control in blue; Mn_EtOAc in red; Co_EtOAc in green) derived from the liquid culture medium of *Aspergillus* sp.

The chromatographic profiles, obtained through LC-ESI-HRMS in positive mode, of the EtOAc phases were obtained from the liquid medium in which the fungus was cultured and from the EtOH extracts of the mycelium. The chromatograms were compared across the experiments involving Mn(II), Co(II), and the control (Czapek medium). This analysis enabled the chemical annotation of secondary metabolites and allowed for an assessment of the influence of these metals on metabolite production, which were subsequently isolated. The comparative total ion chromatogram for pyrophen (**1**), ([M+H]^+^ at *m*/*z* 288.1229; Rt = 5.62 min), with chemical annotation in the EtOAc phases and isolated from the liquid medium cultured with Mn(II), demonstrates that the microorganism optimized the production of 1 when compared with the control medium and the medium supplemented with Co(II). Although pyrophen (**1**) is present in the control, it was observed that the medium containing MnCl_2_ led to optimized production of this metabolite. Figure 3 illustrates the respective chromatogram.

**Figure 4:**
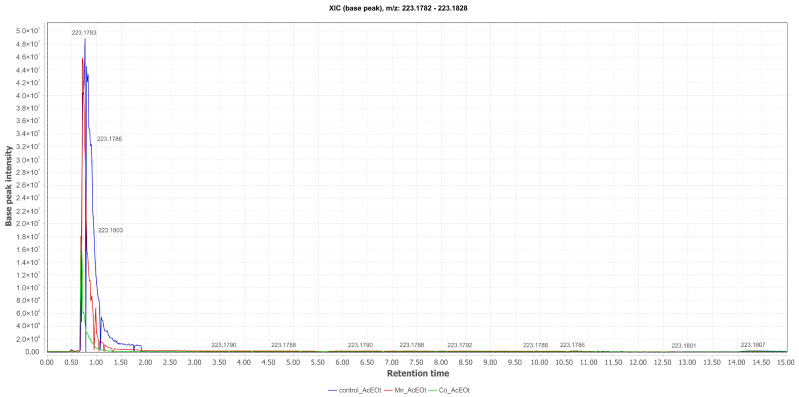
Comparative selected ion chromatograms for nigragillin with *m*/*z* 223.1783 (EtOAc Control in blue; Mn_EtOAc in red; Co_EtOAc in green) derived from the liquid culture medium of *Aspergillus* sp.

Nigragillin (**2**) ([M+H]^+^ at *m*/*z* 223.1783; Rt = 0.77 min) was also annotated in the EtOAc phases and isolated from the liquid medium cultured with Mn(II). The production of this compound was stimulated by the presence of the metal in the fermentation broth, as revealed by the comparative total ion chromatogram between the cultures (Figure 4). However, the production of **2** was optimized in the Czapek medium (control), indicating that this metabolite is not iron-dependent, as it was produced in media containing Mn(II) and Co(II), where iron was suppressed.

**Figure 5:**
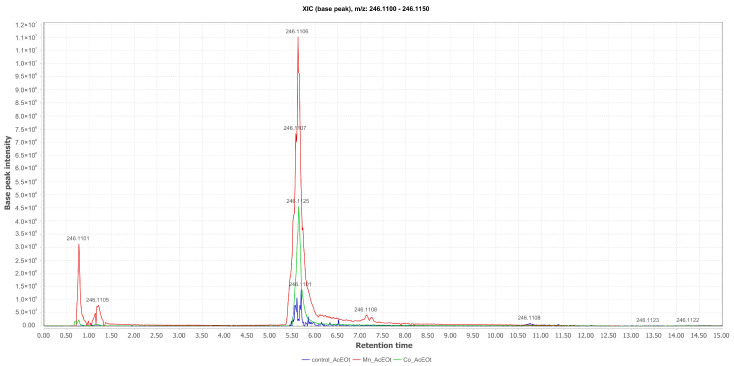
Comparative selected ion chromatograms for penicillquei B with *m*/*z* 246.1125 (EtOAc Control in blue; Mn_EtOAc in red; Co_EtOAc in green) derived from the liquid culture medium of *Aspergillus* sp.

From the analysis of the comparative total ion chromatograms for penicillquei B (**3**) ([M+H]^+^ at *m*/*z* 246.1125; Rt = 5.64 min), obtained from the EtOAc phases and isolated from the liquid medium cultured with Co(II), it was observed that although this substance appears in the control, the presence of CoCl_2_, and particularly MnCl_2_, in the fermentation broth significantly optimized its production (Figure 5).

**Figure 6:**
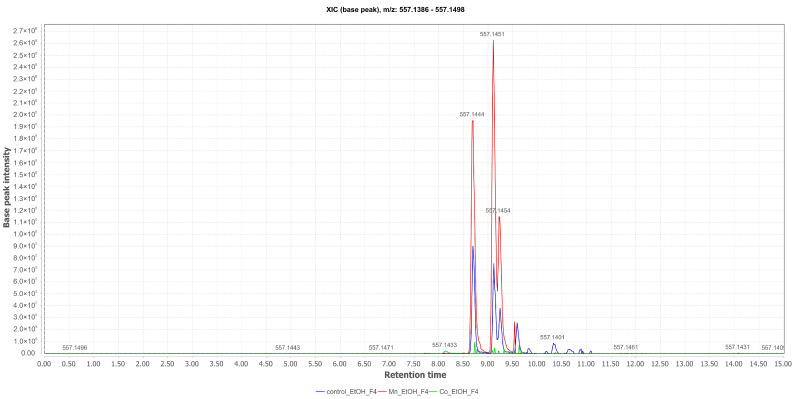
Comparative selected ion chromatograms for aurasperone D with *m*/*z* 557.1451 (EtOAc Control in blue; Mn_EtOAc in red; Co_EtOAc in green) derived from the liquid culture medium of *Aspergillus* sp.

Aurasperone D (**4**) ([M+H]^+^ at *m*/*z* 557.1451 [M+H]^+^; Rt = 9.11 min), isolated from the EtOH fractions of the control obtained from the mycelium, had its production enhanced by the presence of MnCl_2_ in the fungal culture medium. The analysis based on the total ion chromatograms (Figure 5) confirms that the presence of the Mn(II) cation increased the formation of aurasperone D, despite the fact that this substance was not induced, as it also appeared in the control experiment (Figure 6).

**Figure 7:**
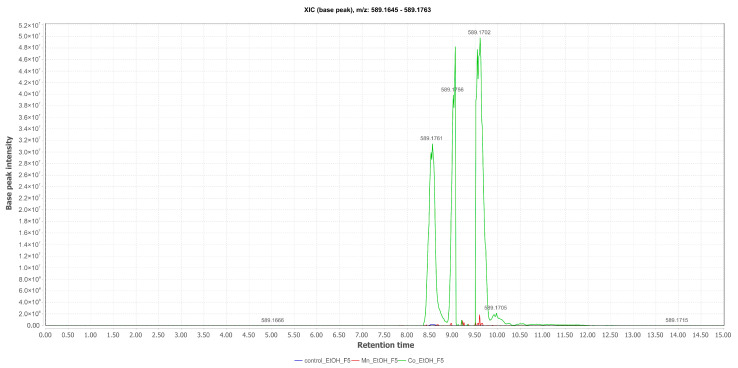
Comparative selected ion chromatograms for Fonsecinone B with *m*/*z* 589.1702 (EtOAc Control in blue; Mn_EtOAc in red; Co_EtOAc in green) derived from the liquid culture medium of *Aspergillus* sp.

Fonsecinone B (**5**) ([M+H]^+^ at *m*/*z* 589.1702; Rt = 9.61 min) was annotated in the EtOH fractions of the mycelium and isolated from the culture with Mn(II), with its production induced by the presence of Co(II) in the medium. The comparative total ion chromatograms between the cultures (Figure 7) reveal that **5** is not a metabolite whose production is dependent on the presence of iron in the medium, as evidence of this substance was also found in the control experiment.

**Figure 8:**
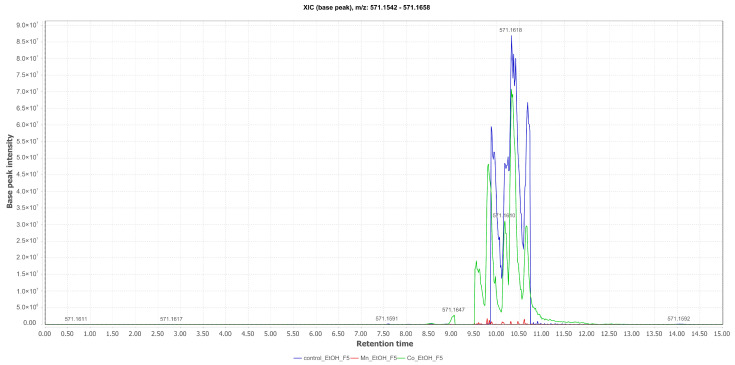
Comparative selected ion chromatograms for fonsecinone A with *m*/*z* 571.1618 (EtOAc Control in blue; Mn_EtOAc in red; Co_EtOAc in green) derived from the liquid culture medium of *Aspergillus* sp.

Fonsecinone A (**6**) ([M+H]^+^ at *m*/*z* 571.1599; Rt = 10.33 min) was annotated in the EtOH fractions of the mycelium and isolated from the culture supplemented with Co(II). This substance had its production optimized when the culture medium was supplemented with CoCl_2_, as compared to the chromatograms of the control medium and the medium supplemented with Mn(II). This suggests that the production of this metabolite is not iron-dependent in the fungal metabolism (Figure 8).

### 2.6. Structural Elucidation of Isolated Substances

**Figure 9:**
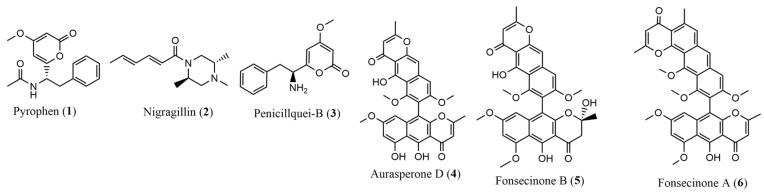
Structural Elucidation of the Isolated Compounds.

The obtained extracts were subjected to a combination of chromatographic processes that led to the isolation of six substances, which were identified based on NMR and HRMS data as shown in Figure 9. From the ^1^H and ^13^C NMR signals, compound **1** was isolated as a white crystalline solid, soluble in MeOD, and identified as pyrophen [31]. Compound **2** was obtained as a yellowish oily substance, soluble in MeOD, and identified as nigragillin [32]. Compound **3**, isolated as a pink amorphous solid, soluble in MeOD, was identified as penicillquei B [21]. Substance **4**, isolated as a yellow amorphous solid, soluble in CDCl_3_, was identified as aurasperone D [33]. Substances **5** and **6** were isolated as yellow amorphous solids, soluble in CDCl_3_, and identified respectively as fonsecinone B [23] and fonsecinone A [34]. The ^1^H and ^13^C NMR signals of the isolated compounds are listed in the Supplementary Materials.

### 2.7. In Vitro Anti-Candida Assays

#### 2.7.1. Activity of Pyrophen, Penicillquei B, and Fonsecinone B Against *C. albicans* and *C. parapsilosis*

Table 2 shows the inhibitory effects of three substances isolated from the endophytic fungus *Aspergillus* sp. against four *Candida* strains. Against standard and clinical strains of *C. albicans*, the pyrophen displayed MIC values for both ranging from 12.39 to 125 µg/mL and MFC values ranging from 39.37 to 280.61 µg/mL, with an MFC/MIC ratio of 1.58–2.24, respectively. Penicillquei B showed the highest detected MIC values (62.5 to 280.61 µg/mL) and MFC values (140.30 to 445.44 µg/mL), with an MFC/MIC ratio of 1.58–2.24 respectively. Fonsecinone B showed the lowest variation in the results for *C. albicans* strains with an MIC of 4.37 to 49.60 µg/mL and MFC of 17.53 to 99.21 µg/mL, with an MFC/MIC ratio of 2–4.01. In the inhibition of *C. parapsilosis* strains, pyrophen and fonsecinone B showed the lowest MIC and MFC values, ranging from 7.80 to 24.79 µg/mL and from 19.68 to 125 µg/mL, respectively, with an MFC/MIC ratio between 2.00 and 5.04. Penicillquei B showed MIC values between 39.36 and 125 µg/mL and MFC between 99.21 and 280.61 µg/mL for the strains tested, with an MFC/MIC ratio ranging from 2.24 to 2.52. Thus, the MIC values found for standard strains of *C. albicans* and *C. parapsilosis* were used as inhibition parameters in in vitro biofilm, toxicity and in vivo infection tests in *T. molitor* larvae.

#### 2.7.2. *C. albicans* and *C. parapsilosis* Biofilm Reduction by Substances Produced by the Endophytic Fungus *Aspergillus* sp.

**Figure 10:**
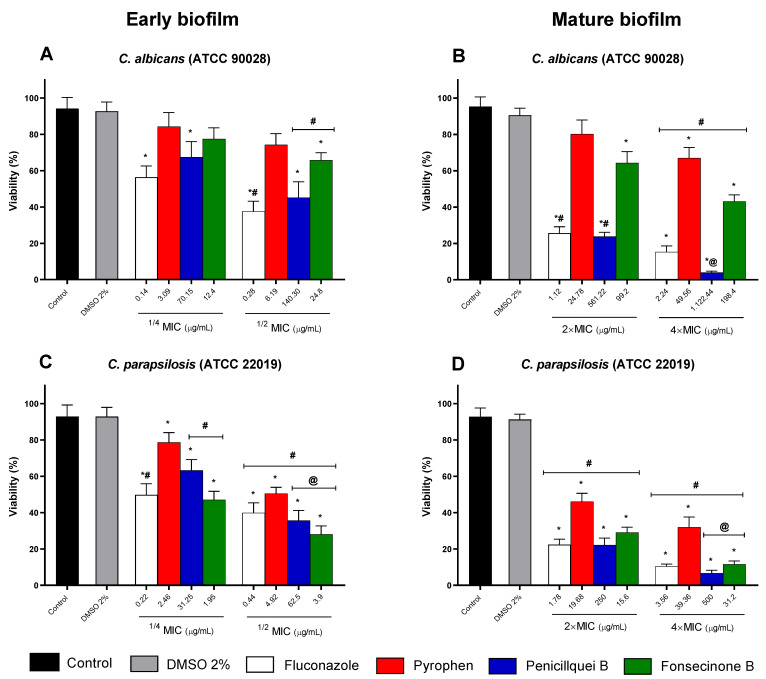
Percentage of inhibition in young (**A**,**C**) and mature (**B**,**D**) biofilms treated with pyrophen, penicillquei B, and fonsecinone B in strains of *C. albicans* (ATCC 90028) (**A**,**B**) and *C. parapsilosis* (ATCC 22019) (**C**,**D**) assessed by the MTT assay. The control is represented by *Candida* strains treated with sterile phosphate-buffered saline (PBS), while 2% dimethyl sulfoxide (DMSO) serves as the solubility agent control for the tested chemical isolates and was designated as a treatment. Treatments were based on the values of 1/4 and 1/2 MIC for forming biofilms and 2× and 4× MIC for mature biofilms and compared with untreated controls or the reference antifungal Fluconazole. (*) *p* < 0.05 compared to control. (#) *p* < 0.005 compared to the other experimental groups. (@) *p* < 0.05 compared to the different concentrations.

Figure 10 shows the viability of *C. albicans* and *C. parapsilosis* biofilms when treated with different concentrations of compounds isolated from the endophytic fungus *Aspergillus* sp. Sub-inhibitory concentrations (Figure 10A,C) proved effective in reducing the viability of forming biofilms, with penicillquei B and fonsecinone B standing out for showing greater reduction at the 1/2 MIC concentration compared to fluconazole (Figure 10C). The reduction profile for concentrations higher than the MIC value (Figure 10B,D) of the isolates is observed across all tested concentrations against the formed biofilm of *C. parapsilosis*, with penicillquei B and fonsecinone B being notably more effective at a concentration of 4× MIC (Figure 10D) compared to fluconazole and untreated controls.

**Figure 11:**
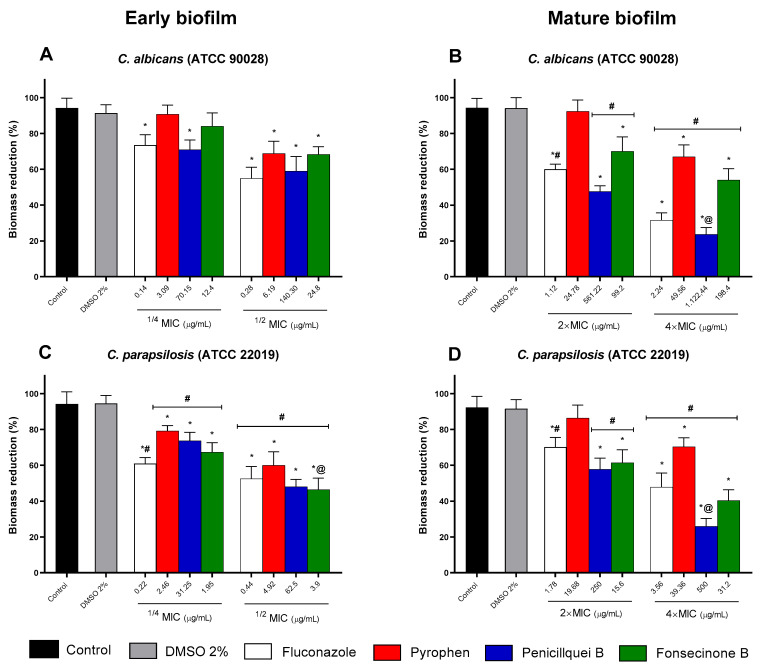
Percentage reduction in biofilm biomass eradication stained with crystal violet for *C. albicans* (ATCC 90028) (**A**,**C**) and *C. parapsilosis* (ATCC 22019) (**B**,**D**). Treatments considered the MIC values (1/4 and 1/2 MIC for forming biofilms and 2× and 4× MIC for mature biofilms) and were compared with untreated controls for each *Candida* strain that received sterile phosphate-buffered saline (PBS) and with a dimethyl sulfoxide (DMSO) group used as solubility agent for the chemical isolates or the reference antifungal Fluconazole. (*) *p* < 0.05 compared to control. (#) *p* < 0.005 compared to other groups. (@) *p* < 0.05 compared different concentration.

The eradication of biofilm biomass of the test strains is depicted in Figure 11 and reveals that concentrations of 1/4 and 1/2 MIC are capable of impairing initial biofilms (Figure 11A,C). However, their effective reduction was better observed when treated with concentrations 4× higher than the MIC, and similar to the effect on viability reduction, penicillquei B was more effective in reducing the biomass of *C. albicans* and *C. parapsilosis* (Figure 11B,D) than the standard drug.

### 2.8. In Vivo Assays

#### 2.8.1. In Vivo Toxicity of Isolated Compounds from *Aspergillus* sp. in *Tenebrio molitor* Larvae

**Figure 12:**
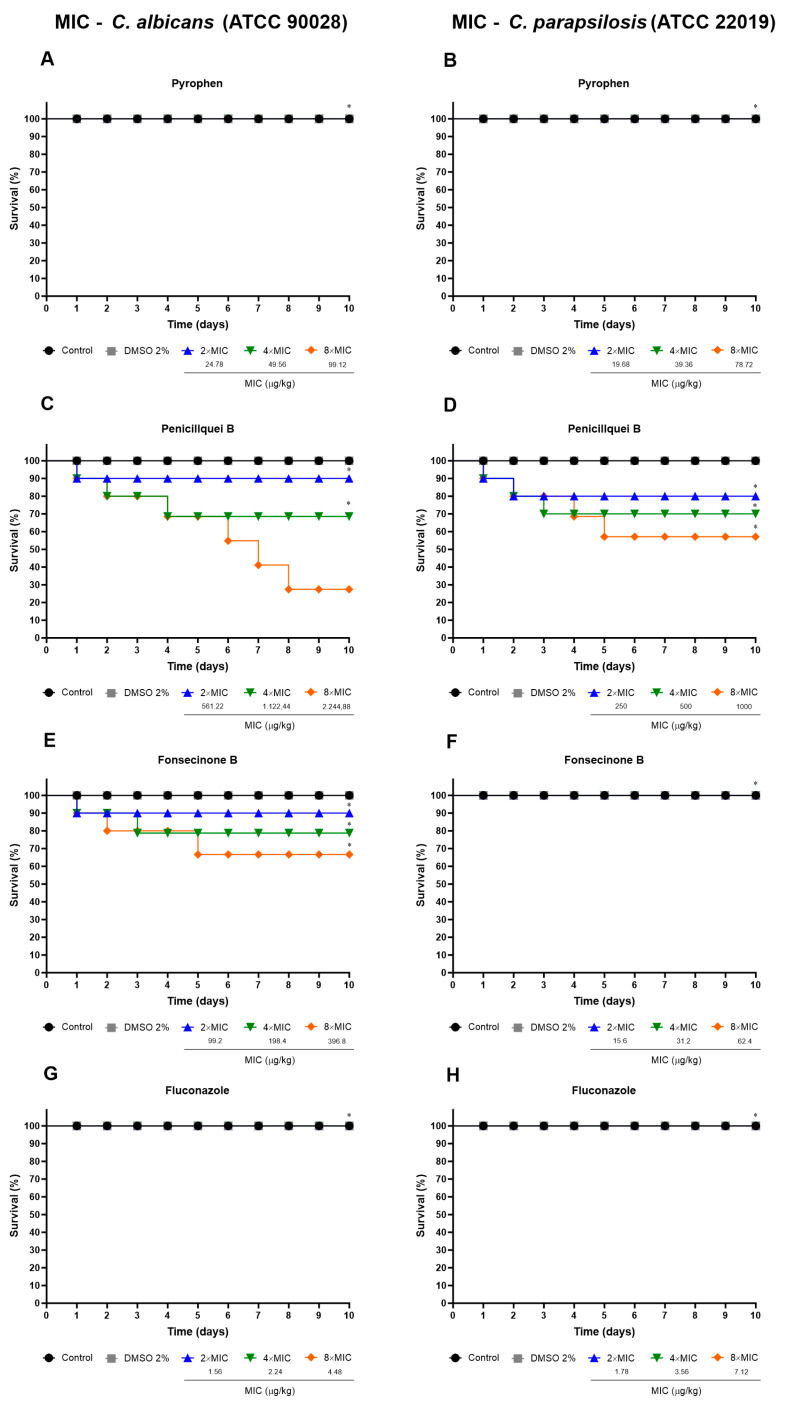
Evaluation of acute toxicity and survival percentage following injection of test substances, pyrophen (**A**,**B**), penicillquei B (**C**,**D**), Fonsecinone B (**E**,**F**), and Fluconazole (**G**,**H**). Treatments were based on concentrations of 2×, 4×, and 8× MIC (µg/kg) for *C. albicans* (ATCC 90028) (**A**,**C**,**E**,**G**) and *C. parapsilosis* (ATCC 22019) (**B**,**D**,**F**,**H**). Controls correspond to *Candida* strains treated with sterile PBS or with dimethyl sulfoxide (DMSO) (2%) as this solubility agent was used for chemical isolates. Survival was assessed using the log-rank test. (*) *p* < 0.05 compared to the control group.

Pyrophen, penicillquei B, and fonsecinone B had their toxicity evaluated on *T. molitor* larvae. The results were encouraging, with low larval mortality. Larvae treated with standard drug fluconazole, pyrophen, and the above-MIC values for *C. parapsilosis* of fonsecinone B achieved 100% survival until the end of the experiment. The mortality rate of larvae treated with 2× MIC/kg concentrations of penicillquei B and fonsecinone B (Figure 12C,E) for *C. albicans* showed survival rates of 90%, with deaths occurring only after the first day of treatment. Penicillquei B at 2× MIC/kg for *C. parapsilosis* presented an 80% survival rate, with deaths occurring up to the second day (Figure 12D). Treatments with 4× MIC/kg showed different variations in mortality rate, with penicillquei B for *C. albicans* showing deaths on the first, second, and fourth day with a survival rate of 68.57% (Figure 12C) and fonsecinone B on the first and third day with a survival rate of 80% (Figure 12E), while deaths with penicillquei B at 4× MIC/kg for *C. parapsilosis* occurred between the first and fourth day with a survival rate of 70% (Figure 12D). The highest percentages of deaths were found at concentrations of 8× MIC/kg, with the values for *C. albicans* related to penicillquei B and fonsecinone B being 24.42% and 66.66% (Figure 12C,E), respectively, while the survival rate found for *C. parapsilosis* of penicillquei B was 57.14% (Figure 12D).

#### 2.8.2. Tolerance of *T. molitor* Larvae Infected with *C. albicans* and *C. parapsilosis* Treated with Pyrophen, Penicillquei Acid B, and Fonsecinone B

**Figure 13:**
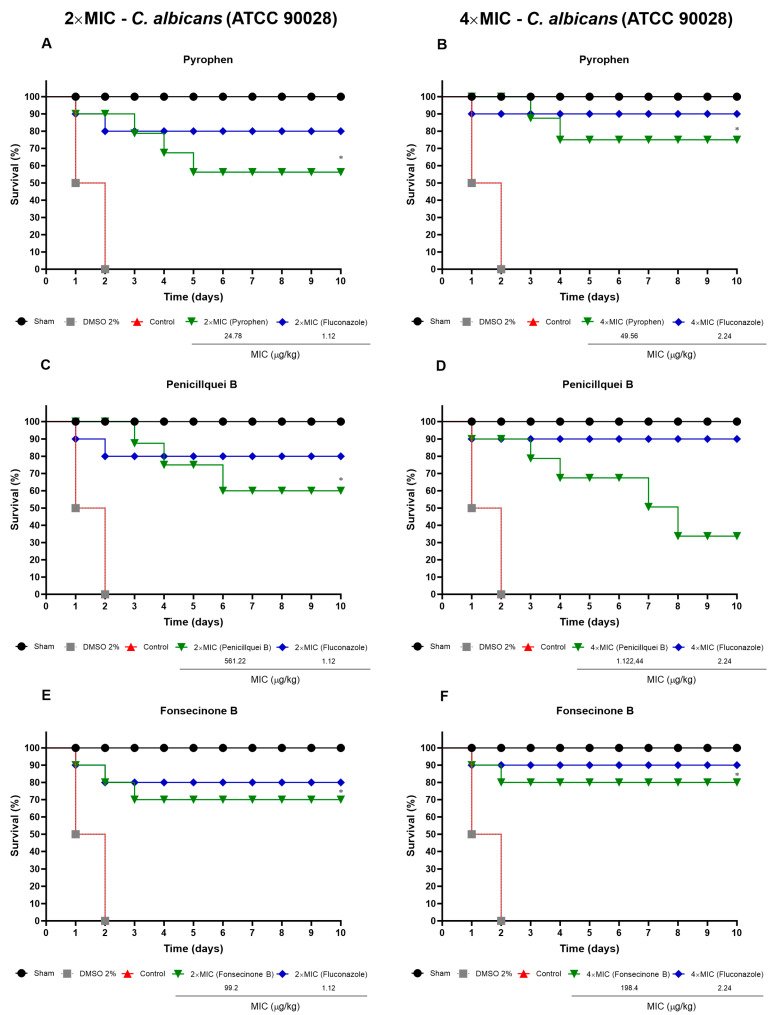
Survival percentage of *T. molitor* larvae after infection with *C. albicans* (ATCC 90028) and treatment at concentrations of 2× and 4× MIC (µg/kg) with pyrophen (**A**,**B**), penicillquei B (**C**,**D**), fonsecinone B (**E**,**F**). Negative controls were treated with sterile PBS or with DMSO (2%), and the positive control was Fluconazole. Survival was assessed using the log-rank test. (*) *p* < 0.05 compared to the control group.

In the treatment using 2× MIC/kg and 4× MIC/kg of pyrophen (Figure 13A,B), survival rates of 56.25% and 75%, respectively, were observed. Penicillquei B at 2× MIC/kg increased survival in 60% of larvae compared to 4× MIC/kg, which showed 33.75% (Figure 13C,D). The treatment with fonsecinone B (Figure 13E,F) achieved the best survival percentages with 70% for treatments at 2× MIC/kg and 80% for 4× MIC/kg, showing similar results to fluconazole. Figure 13 shows the survival rates of *T. molitor* larvae following infection by *C. albicans*.

**Figure 14:**
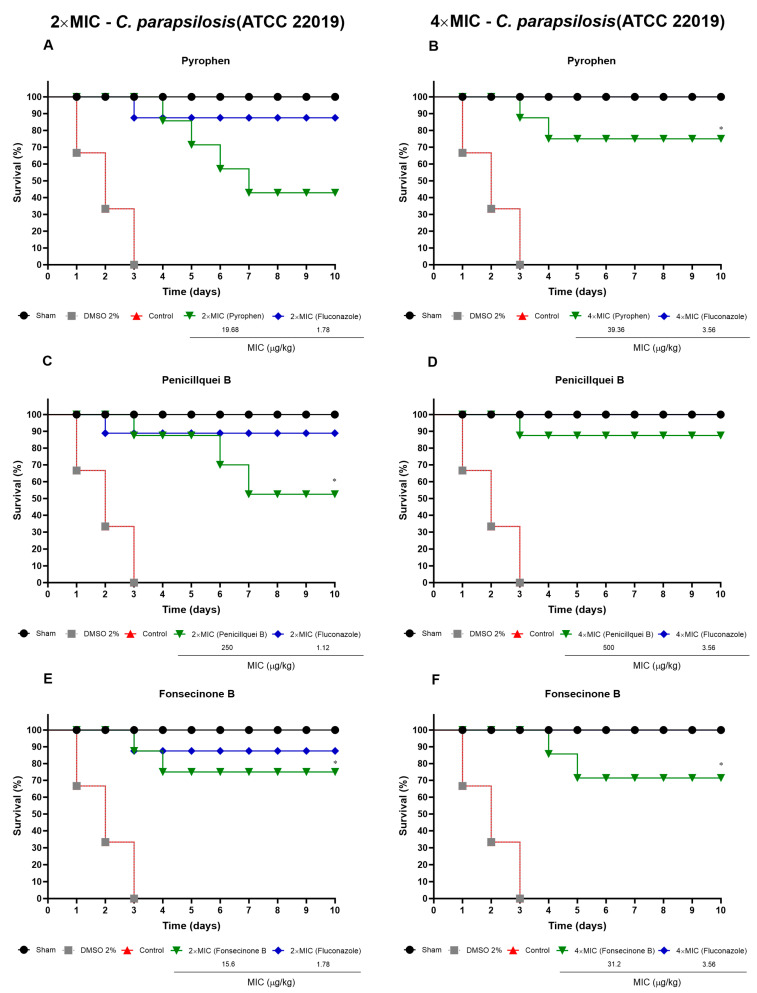
Survival percentage of *T. molitor* larvae after infection with *C. parapsilosis* (ATCC 22019) and treatment at concentrations of 2× and 4× MIC (µg/kg) with pyrophen (**A**,**B**), penicillquei B (**C**,**D**), Fonsecinone B (**E**,**F**). Negative controls were treated with sterile PBS or DMSO (2%), and the positive control was Fluconazole. Survival was assessed using the log-rank test. (*) *p* < 0.05 compared to the control group.

Figure 14 presents the survival of larvae after infection by *C. parapsilosis*, with the best results of the isolates in treatments of 4× MIC/kg (Figure 14B,D,F) ranging from 71.42% to 87.5%.

## 3. Discussion

Based on the observations from the experiments conducted with CoCl_2_ and MnCl_2_ supplementation in the culture medium, distinct chemical profiles were identified, leading to increased production of most of the substances isolated in this study. It is suggested that the presence of Mn(II) and Co(II) cations in the fermentation broth in which *Aspergillus* sp. was cultivated influenced the formation of secondary metabolites, either inducing or suppressing their production when compared to the Czapek control medium.

Metal cations play fundamental roles in the induction and regulation of metabolic pathways, acting as essential cofactors for various enzymes and participating in cellular signaling processes [35,36]. Their presence directly influences the activity of numerous enzymes, enabling reactions that would otherwise not occur or would be inefficient. The Mn(II) and Co(II) cations altered the chemical profile of the endophytic fungus *Aspergillus* sp. due to changes in enzymatic activity, likely attributed to cytochrome P450, a complex formed by an Fe-porphyrin group that participates in redox processes and, consequently, in the formation of secondary metabolites [37,38].

The complexed iron present in the HEME group serves as an enzymatic cofactor in cytochrome P450 monooxygenases. These enzymes are closely involved in the biosynthetic pathways of both primary and secondary metabolites and act as catalysts in various biochemical reactions [39,40]. Therefore, it is proposed that the substitution of the porphyrin core, induced by saturating the *Aspergillus* sp. culture medium with CoCl_2_ and MnCl_2_, significantly alters the catalytic properties and capacities of the enzymes within the fungus, resulting in the different profiles observed between the control and the media containing metal cations.

The compounds were detected both in the fermentation broth and in the more polar fractions of the mycelia. The substances penicillquei B (**3**) and fonsecinone A (**6**) were exclusively identified in the control and in the fraction supplemented with CoCl_2_ salt, respectively, while pyrophen (**1**), nigragillin (**2**), aurasperone D (**4**), and fonsecinone B (**5**) were detected in both cultures.

Nigragillin (**2**) was previously observed in studies with *A. awamori* in medium supplemented with lychee peel powder [20], while aurasperone D (4) was reported in *A. niger* cultured in Czapek medium supplemented with ZnSO_4_ and CuSO_4_ salt solutions [22]. Penicillquei B (**3**) was isolated for the first time in rice culture of the genus *Penicillium* [21].

Compound (**7**), named furancarboxylic acid, was previously identified in the extract of endophytic *A. niger* isolated from the Brazilian coast [24]. Chlovalicin (**8**) was identified in ethanolic extracts reported in the literature [25,34]. Malformin A (**9**) was previously reported in studies conducted by [41] along with other metabolites of the same class (malformin A, B, and C). Epi-aspergillusol (**10**) was also identified by [42]. Asperazine (**11**) was first reported in *A. niger* isolated from marine sponges [28]. Substance (**12**), characterized as hydroxydehydronigerone and considered a derivative of nigerone from the naphthoquinone group, was reported for the first time by [29].

The rising resistance of *Candida* species to conventional antifungals, such as azoles and echinocandins, makes it increasingly necessary to discover new therapeutic agents [13], including the compounds isolated in this study. Pyrophen, penicillquei B, and fonsecinone B, derived from the endophytic fungus *Aspergillus sp*., have shown promising activity against *C. albicans* and *C. parapsilosis*, two leading species responsible for invasive fungal infections. The MIC values for these compounds varied widely, with fonsecinone B displaying the lowest MIC range (4.37–49.60 µg/mL), suggesting a robust inhibitory effect. Penicillquei B, by comparison, demonstrated higher MIC values (62.5–280.61 µg/mL), indicating more moderate activity. The MFC values further support their fungicidal potential, with fonsecinone B presenting the best MFC/MIC ratio, ranging from 2 to 4.01, suggesting an optimal balance between inhibiting and eradicating *Candida* spp.

The compounds also demonstrated highly significant effects against biofilms formed by *C. albicans* and *C. parapsilosis*. Biofilms are structured communities of fungal cells that develop on biological or inert surfaces, and their formation is a major virulence factor of the *Candida* genus [17]. The presence of biofilms is directly linked to increased resistance to antifungal treatments, as cells organized within biofilms possess additional protection against therapeutic agents, enhancing the pathogenicity of *Candida* sp. and complicating infection treatment, particularly in immunocompromised patients [43]. Moreover, biofilms account for a significant proportion of clinical antifungal resistance, contributing to recurrent and hard-to-eradicate infections [13]. At concentrations of 4× MIC, penicillquei B and fonsecinone B proved more effective in reducing biofilm biomass than fluconazole. These findings align with studies highlighting fluconazole’s difficulty in eradicating mature *Candida* biofilms, whose resistance mechanisms involve extracellular matrix formation and the regulation of resistance gene expression [44].

A lower metabolic viability was observed in mature biofilms compared to young biofilms. This effect can be attributed to the greater complexity and density of the polysaccharide matrix present in mature biofilms since those structures create a physical and chemical barrier that hinders antimicrobial penetration [45]. The dense matrix restricts access to nutrients and oxygen, resulting in low-metabolism regions and cells in a dormant state, making mature biofilms more resistant to treatments [46]. Furthermore, cells in the inner layers of mature biofilms exhibit reduced metabolic activity, decreasing the efficacy of antimicrobials that rely on active metabolic processes to target cells. The ability of the compounds under investigation to reduce both biofilm viability and biomass underscores their potential as multifunctional therapeutic agents, targeting different stages of biofilm formation and maturation.

Studies on the use of endophytic fungi against *Candida* biofilms are limited; however, some results suggest that the biofilm inhibition is associated with the ability to modify cell morphology and inhibit cell adhesion to surfaces, one of the initial steps in biofilm formation. For instance, Lim et al. [47] investigated the extract of *Phomopsis longicolla* and reported a 60% reduction in *C. albicans* biofilm formation within 48 h at a concentration of 50 µg/mL. The study also revealed that the extract induced morphological alterations in *Candida* cells, decreasing hyphae formation—a critical process for virulence and biofilm structure. This morphological effect suggests a potential for modulating yeast cell physiology, which may contribute to inhibiting biofilm formation and biomass maintenance.

However, the effects of endophytic fungal extracts are not limited to merely reducing biofilm biomass. Liao et al. [48] demonstrated that extracts from *Pestalotiopsis microspora* reduced *C. albicans* biofilm biomass by up to 68% at concentrations of 100 µg/mL. Additionally, these extracts reduced the production of extracellular polysaccharides—essential components of the biofilm matrix—by up to 52%.

The efficacy of endophytic fungal extracts against *Candida* biofilms is also linked to the composition and diversity of bioactive compounds present in these extracts. Studies have identified various substances, including alkaloids, flavonoids, fatty acids, and terpenes, as responsible for antifungal activity. You et al. [49] found that extracts of *Trichoderma harzianum* inhibited biofilm formation by up to 75% and reduced cell viability by 50% at a concentration of 150 µg/mL. This research underscores the importance of exploring the chemical diversity of endophytic fungi to develop novel antifungal and antibiofilm agents.

The use of *Tenebrio molitor* larvae as an efficient in vivo model for toxicity studies of antifungal substances is well-established, and it has been shown that the compounds pyrophen, penicillquei B, and fonsecinone B exhibited moderate toxicity, with survival rates exceeding 80% at doses of up to 2× MIC/kg. The highest mortality was observed with penicillquei B at 8× MIC, where the survival rate for *C. parapsilosis* was only 57.14%.

The findings presented here are promising, particularly as penicillquei B had not previously been experimentally evaluated for any biological activity. This study represents the first report of its biological activity. Pyrophen has been previously evaluated for its anti-*Candida* activity, yielding exciting results [50]. While the previous study assessed the same strain, our findings are more robust, as we employed the broth microdilution method, whereas the earlier research used the agar well diffusion method. Our methodology ensures better contact between the compound and the pathogen and allows for a more thorough qualitative and quantitative analysis. Additionally, we evaluated the compound’s effects on biofilm formation, toxicity, and survival.

There are few reports on the activities of these compounds. Fonsecinone B has been documented to exhibit 36.9% DPPH radical-scavenging activity at a concentration of 250 μg/mL and showed immunomodulatory potential in vitro, with a 35% reduction in cell viability in RAW264.7 macrophages when tested at 100 μg/mL [51]. Although fonsecinone A was not experimentally evaluated in this study, it was identified and isolated from our extracts and is well-documented in the literature for its high biological activity, including antibacterial [52], antifungal [53], anti-inflammatory [34] properties, as well as its enhanced acetylcholinesterase inhibitory activity when synthesized with silver nanoparticles [54]. The present results are encouraging and a guide for future directions of the study, including investigations in vivo, evaluation of molecular mechanisms involving both cation-regulated genes and anti-*Candida* activity, development of formulations to enhance bioavailability and stability, as well as studies on synergism and biotechnological applications of these metabolites.

## 4. Materials and Methods

### 4.1. Endophytic Fungus

The original fungal strain of *Aspergillus* sp. under code MA-O3 was obtained from the culture collection of the Federal Institute of Maranhão. It was isolated from the leaves of *Dizygostemon riparius* (Plantaginaceae) collected in the municipality of São Benedito do Rio Preto (3°20′02″ S 43°31′40″ W), Maranhão, BR, in 2019.

### 4.2. Fermentation and Extraction

The *Aspergillus* sp. fungal strain was reactivated on a PDA plate at 25 °C for 7 days. The inoculum was prepared individually from 0.5 × 0.5 cm cuts in three different cultivation conditions. In the first condition, the fungus was cultivated in 20 × 500 mL Erlenmeyer flasks containing 150 mL of liquid Czapek medium composed of NaNO_3_ (3.0 g L^−1^); K_2_HPO_4_ (1.0 g L^−1^); MgSO_4_ (0.5 g L^−1^); KCl (0.5 g L^−1^); FeSO_4_‧7H_2_O (0.01 g L^−1^); glucose (30.0 g L^−1^); and yeast extract (20.0 g L^−1^). The second and third conditions were similar, but with the addition of MgCl_2_ (3.1 g L^−1^) and CoCl_2_ (3.21 g L^−1^), respectively. The fermentation was maintained for 21 days under static conditions at room temperature.

At the end of the fermentation for each condition, the culture broth was separated from the fungal mycelium by partitioning with ethyl acetate (1:1), resulting in an organic phase, which was dried in a rotary evaporator under low pressure, obtaining the EtOAc phases (F.EtOAc) from the three cultivation conditions: control (Czapek, Geneva, Switzerland), MnCl_2_, and CoCl_2_, named respectively as CA, MnA, and CoA. The mycelium was subsequently extracted with EtOH (150 mL) and left to rest for 24 h, followed by filtration and evaporation of the solvent under reduced pressure, producing the crude ethanolic extracts of the control (Czapek), MnCl_2_, and CoCl_2_, named respectively as CM, MnM, and CoM. After obtaining the organic phases in EtOAc and the extracts in EtOH, their chemical profiles were evaluated by CCDC and UHPLC.

### 4.3. Analysis by LC-ESI-HRMS for EtOAc Phases and EtOH Extracts

Chemical analysis was performed for the EtOAc (CA, MnA, and CoA) and EtOH (CM, MnM, and CoM) extracts using LC-ESI-HRMS with liquid chromatography on a C18 column (100 mm × 2.1 mm × 2.6 mm). The binary mobile phase was composed of H_2_O (A) and CH_3_CN (C), while MeOH (B) was used to clean the column at the end of each run. The elution gradient started with 5% mobile phase B (0.1% formic acid in acetonitrile), increasing linearly to 40% B in 5 min, followed by an increase to 45% B in 2 min, and reaching 98% B in 18 min, where it was held for 2 min. In the final minutes (13 to 15), 100% solvent B was used to ensure complete cleaning of the column. After this, the phase was reverted to 95% A (0.1% formic acid) in 2 min, being maintained until minute 24. Mass spectrometry data were acquired with electrospray ionization (ESI) in positive mode, with a scan range of *m*/*z* 100 to 1500 Da.

### 4.4. Fractionation and UHPLC-UV Analysis of Fractions Obtained from EtOAc Phases and EtOH Extracts

The EtOAc phases (CA, MnA, and CoA) were subjected to column chromatography (CC) under gravity, packed with silica gel 60 (0.040–0.063 mm), using a polarity gradient elution system with Hex/EtOAc (7:3) and EtOAc/MeOH (9:1, 3, 5, 10, 15, 20, 50, 80, and 100%) to produce 12 fractions from each cultivation mode (CAF1 to CAF12 for CA, MnAF1 to MnF12 for MnA, and CoAF1 to CoF12 for CoA). The crude EtOH extracts were also fractionated by CC under the same conditions, but with a polarity gradient system of Hex/EtOAc (9:1, 1:1, and 4:6), EtOAc/MeOH (9:1, 1:1), and 100% MeOH, resulting in 6 fractions from each cultivation mode named CMF1 to CMF6 for the control, MnMF1 to MnMF6 for MnM, and CoMF1 to CoMF6 for CoM.

The fractions obtained from ethyl acetate extractions (F.AcOEt) and ethanol extractions (E.EtOH) were subjected to ultra-high-performance liquid chromatography (UHPLC) analysis in gradient mode with reverse phase. For the analysis of the crude EtOAc extracts and the fractions obtained from the EtOH extract, a UHPLC-DAD method was used, with a solid-core stationary phase column (AcQUITY UPLC CSR C18 1.7 μm, 100 mm × 2.1 mm). The binary mobile phase was composed of H_2_O (A), CH_3_CN (C), and MeOH (B), the latter used to clean the column. The analysis was performed over 15 min, starting with a flow rate of 0.400 mL/min and a composition of 85% H_2_O (A), 0% MeOH (B), and 15% CH_3_CN (C), maintained until minute 1.0. At 4.0 min, the solvent ratio was adjusted to 70% H_2_O (A), 0% MeOH (B), and 30% CH_3_CN (C). Then, at 9.0 min, the mobile phase was changed to 30% H_2_O (A), 0% MeOH (B), and 70% CH_3_CN (C). At 11.0 min, the composition was changed to 100% CH_3_CN (C) while maintaining a flow rate of 0.400 mL/min. From 11.2 to 12.5 min, the flow rate was adjusted to 0.500 mL/min, using 100% MeOH (B) for the column-cleaning step. Finally, from 13.0 to 15.0 min, the flow rate was reduced again to 0.400 mL/min, with 90% H_2_O (A), 0% MeOH (B), and 10% CH_3_CN (C), allowing the column to be reconditioned before the next analysis. This UHPLC-DAD method enabled efficient separation of the compounds, with specific elution, cleaning, and reconditioning steps to ensure optimal analysis conditions.

### 4.5. Isolation and Structural Determination by NMR

The combined fractions CAF_5 to 8, MnAF_10 to 12, and CoAF_6 to 8, originating from the EtOAc phases (CA, MnA, and CoA), respectively, were purified by preparative recyclable high-performance liquid chromatography (RP-HPLC). This was conducted using an Asahipak GS-310 (13 µm, 20 × 500 mm)-Sephadex polyvinyl column on a Shimadzu SCL-10Avp system equipped with Shimadzu LC-6AD gradient pumps and a Shimadzu SPD-10 vvp UV detector with a recycle valve (100% MeOH at 3.0 mL/min) and UV-vis detection at 280 to 325 nm with isocratic elution in MeOH. This process yielded the compounds pyrophen (**1**), nigragillin (**2**), and penicillquei B **(3**).

Fractions CMF4, MnMF5 to MnMF6, and CoMF5, obtained from the EtOH extracts, were further fractionated by CC on silica gel 60 (0.040–0.063 mm) with a Hex/EtOAc (7:3) and MeOH (9:1 to 100%) elution system, generating 12 sub-fractions for each selected fraction. Subsequently, the combined sub-fractions CMF4_5 to 7, MnMF5-6_7, and CoMF5_5 to 7 were subjected to preparative HPLC using a Shimadzu system equipped with an SCL-10Avp, a C-18 column (1.0 µm, 250 mm × 21.20 mm), MeOH and H_2_O as the mobile phase, a flow rate of 8.0 mL/min, and UV-vis detection set at 235 to 325 nm, with gradient elution (MeOH/H_2_O 9:1). This resulted in the compounds aurasperone D (**4**), fonsecinone B (**5**), and fonsecinone A (**6**).

The ^1^H NMR, ^13^C NMR spectra, and 2D experiments, such as gradient-selected heteronuclear multiple bond coherence (HMBC (^1^H/^13^C)), were recorded on a Bruker Avance III spectrometer operating at 400 MHz. TMS was used as an internal standard. This procedure was performed on a 9.4 Tesla NanoBay, equipped with an automatic sample changer, a 5 mm BFO smart probe with ATMA^®^, and a *Z*-axis field gradient coil.

### 4.6. In Vitro and In Vivo Tests for Anti-Candida Activity

#### 4.6.1. *Candida* Strains, Growth Conditions, and Inoculum Preparation

Standard strains of *C. albicans* (ATCC 90028) and *C. parapsilosis* (ATCC 22019), along with clinical isolates from blood infections of both species, were sourced from the collection of the Laboratory of Immunophysiology at the Federal University of Maranhão, São Luís, MA, Brazil. The yeasts were reactivated on Sabouraud dextrose agar (Kasvi, Italy) for 24 h at 37 °C. The inoculum was prepared in a NaCl (0.85%) solution from colonies cultured for 24 h and adjusted using spectrophotometry (Global Trade Technology) at a wavelength of 530 nm to a cell density equivalent to McFarland standard 0.5. For the microdilution tests, the suspension was diluted in RPMI 1640 with glutamine, without bicarbonate (Sigma-Aldrich, St. Louis, MO, USA), pH 7.0, and buffered with 3-(N-morpholino) propane-sulfonic acid (MOPS, Sigma Chemical, St. Louis, MO, USA) at a concentration of 1 × 10^3^ to 5 × 10^3^ CFU/mL [55].

#### 4.6.2. Determination of Minimum Inhibitory Concentration (MIC)

The susceptibility profile of *C. albicans* and *C. parapsilosis* test strains to the chemical isolates pyrophen, penicillquei B, and fonsecinone B was standardized following criteria established by the Clinical and Laboratory Standards Institute (M27-A3) [55], with adaptations. For the assay, 100 µL of RPMI 1640 medium (with glutamine and phenol red, without bicarbonate) buffered with 3-(N-morpholino) propane-sulfonic acid (MOPS) was added to the wells of a sterile 96-well microtiter plate, which contained the standardized *Candida* inoculum suspensions. Subsequently, 100 µL of the chemical isolates at an initial concentration of 1000 µg/mL were added to the first column, followed by serial dilutions down to 1.953 µg/mL. Fluconazole (0.125–64 µg/mL) (Sigma-Aldrich, São Paulo, Brazil) was used as a positive control. RPMI 1640, without the test substances, was used as a negative growth control, and 2% (*v*/*v*) DMSO was used as a vehicle control. The assay microplates were incubated for 48 h at 37 °C, and the outcome was visually analyzed and read on a microplate reader at 540 nm. The MIC was defined as the lowest concentration of the extract or antifungal agent at which no growth was visible or detected. The test was performed in triplicate in two independent experiments.

#### 4.6.3. Determination of Minimum Fungicidal Concentration (MFC)

The MFC was determined based on the results found in the MIC assay. A 10 μL aliquot from the wells corresponding to up to 4× the MIC value was transferred to Petri dishes containing Sabouraud dextrose agar with chloramphenicol (Kasvi, Italy) [18]. The plates were incubated for 24 to 48 h at 37 °C, and the MFC was defined as the lowest concentration of the test substances that inhibited fungal growth in colonies. All tests were performed in triplicate in two independent experiments. The MFC/MIC ratio was calculated to determine whether the extract had fungistatic (MFC/MIC ≥ 4) or fungicidal (MFC/MIC ≤ 4) activity [56].

#### 4.6.4. Effect of Pyrophen, Penicillquei B, and Fonsecinone B on *Candida* Biofilms

The assays were conducted according to the inoculum standardization protocol described previously. Following this, the inoculum was cultivated in yeast nitrogen base (YNB; Sigma-Aldrich) supplemented with 50 mM glucose and incubated at 37 °C for 18 h. The yeast suspensions were centrifuged at 1666× *g* (Centrifuge 5810R, Eppendorf) for five minutes, and the cell pellet was washed twice with sterile PBS and then resuspended in 5 mL of sterile PBS. The optical densities of the inocula were adjusted to 1 × 10^7^ CFU/mL. Moreover, 200 µL of each yeast suspension was transferred to 96-well microtitulation plates (Kasvi, Ortygia) and incubated for 90 min at 37 °C for the adhesion process. Then, the supernatants were aspirated, and the wells were washed twice with sterile PBS to remove non-adherent yeasts [16,57].

To evaluate the inhibitory effects of the compounds under study on young (preformed) biofilms, 200 µL of each compound at sub-inhibitory concentrations of 1/4 MIC and 1/2 MIC, diluted in YNB supplemented with 50 mM glucose, was added to each well with adherent cells, followed by incubation for 24 h at 37 °C. After incubation, the supernatant was aspirated, the formed biofilms were washed twice with sterile PBS, and then their viability was determined by the 3-(4,5-dimethylthiazol-2-yl)-2,5-diphenyltetrazolium bromide (MTT) assay (Sigma-Aldrich, St. Louis, MO, USA) and their biomass was defined by crystal violet staining; both methodologies are described in subsequent sections.

In the analysis of inhibitory effects on formed biofilms, 200 µL of each compound at concentrations above the MIC (2× MIC and 4× MIC) diluted in YNB supplemented with 50 mM glucose was added to each well with adherent cells, followed by incubation for 48 h at 37 °C. After incubation, the supernatant was aspirated, the formed biofilms were washed twice with sterile PBS, and then their viability was determined by the MTT assay and their biomass defined by crystal violet staining; both methodologies are described in subsequent sections.

For the analysis of inhibitory effects on mature (formed) biofilms, the inocula were incubated in microplates containing YNB supplemented with 50 mM glucose for 48 h, with the medium being changed at the 24 h mark. Afterwards, the supernatants were aspirated, the biofilms were washed twice with sterile PBS, and 200 µL of the test substances at concentrations above the MIC (2× MIC and 4× MIC) were added. The microplates were incubated for an additional 24 h at 37 °C. After the incubation period, the supernatants were aspirated, and the biofilms were washed twice with sterile PBS and evaluated by MTT and crystal violet staining. In all experiments, biofilms without test substances and biofilms with 2% DMSO were used as negative controls, and fluconazole was used as a positive control. For both assays, triplicates were performed in two independent experiments.

##### Biofilm Viability Assay

The metabolic activity of fungal cells within the biofilm was determined using the MTT method [58], with some modifications. After the washing process of the biofilms, 100 µL of MTT solution (5 mg/mL; Sigma, USA) in YNB medium supplemented with 50 mM glucose was added to each sample, which was then incubated at 37 °C for 4 h in the dark. Subsequently, the supernatants were removed. Then, 100 µL of DMSO was added to each well, and the samples were incubated for an additional ten minutes. The absorbance of the plate was read on a microplate reader (Softmax^®^ Pro; San Jose, CA, USA) at 570 nm.

##### Crystal Violet Staining Analysis of Biofilm

Biofilms were quantified using the crystal violet staining method [59]. After washing the biofilms with sterile PBS, they were air-dried at room temperature and fixed and resuspended in 200 µL of 95% (*v*/*v*) methanol, then incubated for 15 min at 37 °C. The methanol was completely removed, and the plates were air-dried for 20 min at room temperature. Subsequently, 200 µL of crystal violet solution (1% *v*/*v*) was added to each well, and the samples were incubated with the dye for five minutes. The plates were washed twice with sterile PBS, and 200 µL of acetic acid (33% *v*/*v*) was added to each well. To obtain absorbance values, 100 µL from each of the sample wells was transferred to a new 96-well microplate and read at 570 nm on the microplate reader.

### 4.7. In Vivo Assay in Tenebrio molitor Larvae

Larvae of *T. molitor* (Coleoptera, Polyphaga: Tenebrionidae) at early stages, 11th to 12th, and weighing approximately 200 mg, were obtained from specialized breeders and then selected based on size similarity and exhibiting no apparent color alterations for use in all experiments. The larvae were placed in sterile Petri dishes for 24 h prior to the experiments for acclimatization and incubated at 37 °C, protected from light. Larvae displaying dark spots or apparent melanization processes were excluded [17,60].

#### 4.7.1. Acute Toxicity Assay in *T. molitor* Larvae

Pyrophen, penicillquei acid B, and fonsecinone B at concentrations of 2×, 4×, and 8× MIC (µg/kg) were injected in 5 µL using a Hamilton syringe (Hamilton, FL, USA) via the intrahemocoelic route, previously cleaned with 70% alcohol, between the fourth and fifth sternites of the lower ventral abdomen of the larvae (*n* = 30 larvae/group). Control groups consisted of larvae that received sterile PBS and 2% DMSO. Subsequently, the larvae were incubated at 37 °C, and deaths were assessed every 24 h for 10 days. Death was defined as the complete loss of movement and absence of response to physical stimuli and an intense melanization process [17]. The assays were repeated three times in two independent experiments.

#### 4.7.2. Efficacy of Pyrophen, Penicillquei B, and Fonsecinone B in *T. molitor* Larvae Infected with *C. albicans* and *C. parapsilosis*

Standard strains of *C. albicans* (ATCC 90028) and *C. parapsilosis* (ATCC 22019) were cultivated on Sabouraud dextrose agar (Kasvi, Italy) for 24 h at 37 °C. The inocula were standardized according to Silva et al. [17], with a concentration of 5 × 10^5^/5 µL considered lethal. For this assay, treatment concentrations corresponding to 2× MIC and 4× MIC (µg/kg) were used, relating to the inhibition of the standard strains by the chemical isolates and fluconazole. The larvae (n = 30 larvae/group) received 5 µL of the standard inoculum via the previously described route and region, then were placed in Petri dishes and incubated at 37 °C. After a period of 3 h, the larvae received a single dose of 5 µL of the test concentrations, and the plates were again incubated at 37 °C. The control groups corresponded to sham (larvae without intervention), control (infected larvae treated with sterile PBS), and 2% DMSO (infected larvae treated with 2% DMSO). Mortality rates for each group were determined every 24 h for 10 days, with deaths evaluated by the same criteria previously mentioned. The assays were repeated three times in two independent experiments.

### 4.8. Statistical Analysis

The GraphPad Prism 9.0 software (La Jolla, CA, USA) was used for graphical and statistical purposes. Results are expressed as mean ± standard deviation. Statistical analysis was performed using the Student’s *t*-test. Survival curves were analyzed using the log-rank test. A *p*-value < 0.05 was considered statistically significant.

## 5. Conclusions

The addition of metal cations, such as Mn(II) and Co(II), to the culture medium of endophytic *Aspergillus* sp. isolated from *Dizygostemon riparius* significantly influenced the production of secondary metabolites with antifungal activity. Notably, pyrophen, penicillquei B, and fonsecinone B exhibited promising activity against *Candida albicans* and *C. parapsilosis*, with fonsecinone B standing out due to its lower MIC values and a favorable MFC/MIC ratio, indicating strong inhibitory and fungicidal potential. Moreover, fonsecinone B effectively reduced both the viability and biomass of biofilms formed by these *Candida* species, surpassing the efficacy of fluconazole in some cases. Toxicity assays demonstrated that these compounds exhibit moderate toxicity, suggesting they can be considered relatively safe at concentrations up to 2× MIC/kg. These findings, combined with the observed antifungal and antibiofilm activities, suggest that these metabolites hold promise as multifunctional therapeutic agents.

Future research should focus on more in-depth in vivo studies to assess safety in mammals, investigation into the molecular mechanisms underlying both the influence of metal ions on the culture medium and the effects of the compounds on the fungus, synergy studies with standard antifungal drugs, and the development of formulations aimed at optimizing the bioavailability and stability of these compounds for therapeutic use.

## Figures and Tables

**Table 1:** Compounds annotated by UPLC-UV-DAD in positive mode in the EtOAc phases (CA, MnA, and CoA) and the extracts (CM, MnM, and CoM) obtained from *Aspergillus* sp.

Nª	RT (min)	Calculated Mass (Da) [M+H]^+^	Observed Mass (Da) [M+H]^+^	Annotated	EtOAc Phase (CA)	EtOAc Phase (MnA)	EtOAc Phase (CoA)	EtOH Fraction (CM)	EtOH Fraction (MnM)	EtOH Fraction (CoM)	Ref
1	5.62	288.1230	288.1231	pyrophen	X	-	--	--	--	--	[19]
2	0.72	223.1805	223.1804	nigragillin	--	X	--	--	--	--	[20]
3	5.64	246.1125	246.2425	penicillquei B	--	--	X	--	--	--	[21]
4	9.72	557.1442	557.1436	aurasperone D	--	--	--	X	--	--	[22]
5	9.6	589.1704	589.1705	fonscecinone B	--	--	--	--	X	--	[23]
6	10.34	571.1599	571.1599	fonsecinone A	--	--	--	--	--	X	[23]
7	5.65	288.1230	288.1229	furancarboxylic acid	X	--	--	--	--	--	[24]
8	5.89	332.1390	332.1391	chlovalicin	--	--	--	--	--	X	[25]
9	8.01	530.2465	530.2463	malformin A1	X	--	--	--	--	--	[26]
10	6.09	247.0965	247.0977	epi-aspergillusol	--	X	--	--	--	--	[27]
11	6.95	665.2871	665.2863	asperazine	--	X	--	--	--	--	[28]
12	9.54	589.1704	589.1716	2-hydroxydihydronigerone	--	--	--	--	X	--	[29]
13	10.22	571.1599	571.1600	nigerone	--				X		[30]

Retention time (RT); minutes (min); acetate phase control (CA); acetate phase manganese (MnA); acetate phase cobalt (CoA); ethanol fraction control (CM); ethanol fraction manganese (MnM); ethanol fraction cobalt (CoM); X = annotated compound; -- = unannotated compound.

**Table 2:** Minimum Inhibitory Concentration (MIC) and Minimum Fungicidal Concentration (MFC) of natural products produced by the endophytic fungus *Aspergillus* sp.

	Isolated from the Endophytic Fungus *Aspergillus* sp.									Antifungal
	Pyrophen			Penicillquei B			Fonsecinone B			FLUCO
*Candida* Strain	MIC ^a^	MFC ^a^	MFC/MIC Ratio	MIC	MFC	MFC/MIC Ratio	MIC	MFC	MFC/MIC Ratio	MIC
*C. albicans* (ATCC 90028) ^b^	12.39	39.37	3.17	280.61	445.44	1.58	49.60	99.21	2.00	0.56
*C. albicans* (clinical) ^c^	125	280.61	2.24	62.5	140.30	2.24	4.37	17.53	4.01	1.12
*C. parapsilosis* (ATCC 22019) ^b^	9.84	44.19	4.49	125	280.61	2.24	7.80	15.61	2.00	0.89
*C. parapsilosis* (clinical) ^c^	7.80	19.68	2.52	39.36	99.21	2.52	24.79	125	5.04	1.41

^a^ Values are expressed in µg/mL. ^b^ American Type Culture Collection (ATCC^®^). ^c^ Clinical strain.

## Data Availability

No new data were created or analyzed in this study.

## Supplementary Materials

The following supporting information can be downloaded at: https://www.mdpi.com/article/10.3390/ph17121678/s1, Figure S1: (A) Comparative Chromatograms Obtained from UHPLC-DAD of Fractions CAF4, MnAF4, CoAF4, BCAMF4, BMnAMF4, and BCo-AMF4; (B) Comparative chromatograms obtained from UHPLC-DAD of fractions CAF5, MnAF5, CoAF5, BCAMF5, BMnAMF5, and BCoAMF5; and (C) Comparative Chromatograms Obtained from UHPLC-DAD of Fractions CAF6, MnAF6, CoAF6, BCMF6, BMnMF6, and BCoMF6; Table S1: NMR Data (1H and 13C) in MeOD for the Substances: Pyrophen (1), Nigragillin (2), Penicillquei B (3), Aurasperone D (4), Fonsecinone B (5), and Fonsecinone A (6).
