# Supplementary material for: Effect of Mn(II) and Co(II) on Anti-Candida Metabolite Production by Aspergillus sp. an Endophyte Isolated from Dizygostemon riparius (Plantaginaceae)
Source: Pharmaceuticals (Basel). 2024 Dec 12;17(12):1678. doi: 10.3390/ph17121678 (PMC11677262; doi:10.3390/ph17121678)
Supplement: Supplementary file 1 [file pharmaceuticals-17-01678-s001.zip › pharmaceuticals-3336038-supplementary.pdf]

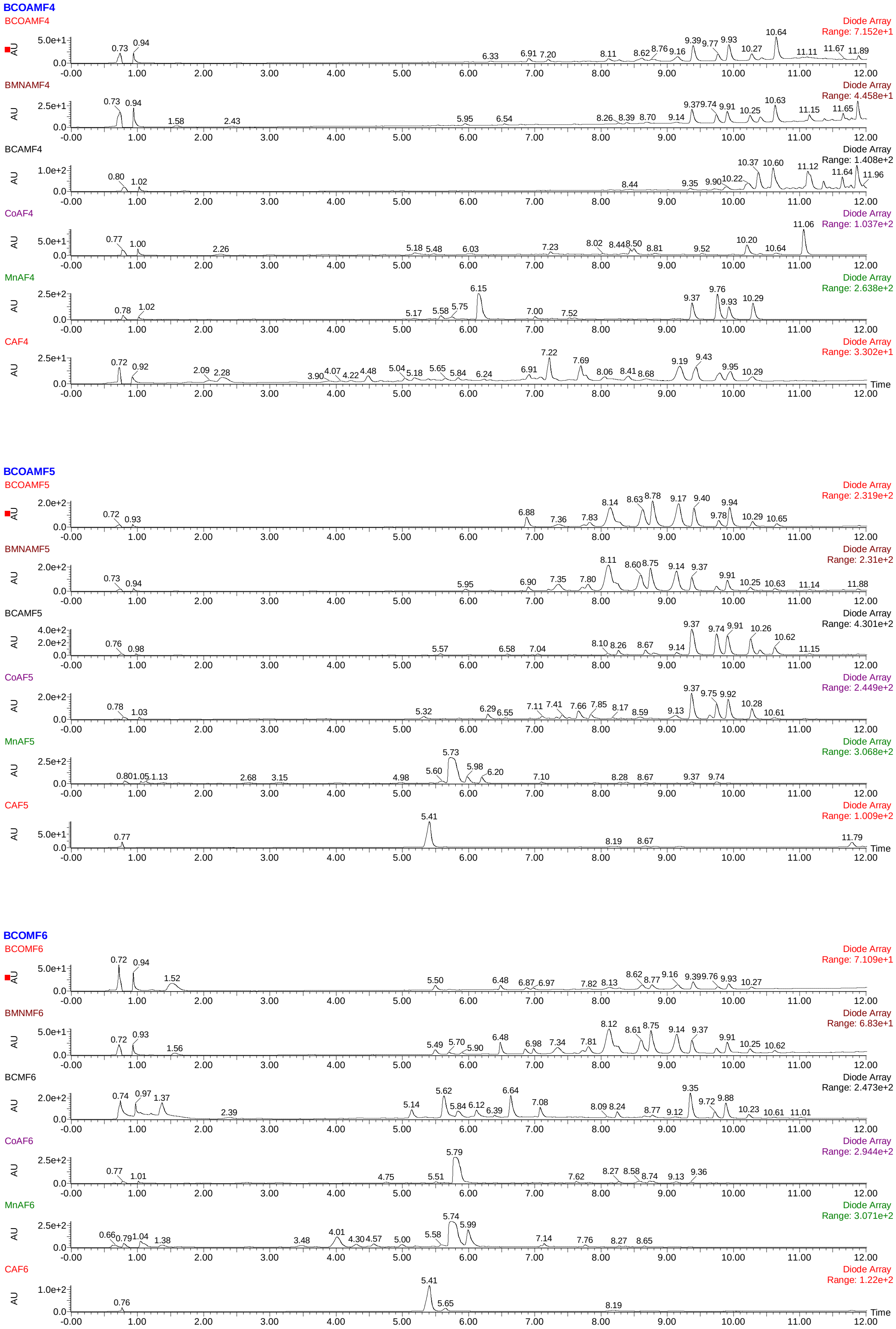

Figure MS1. (A) Comparative Chromatograms Obtained from UHPLC-DAD of Fractions CAF4, MnAF4, CoAF4, BCAMF4, BMnAMF4, and BCo-AMF4; (B) Comparative chromatograms obtained from UHPLC-DAD of fractions CAF5, MnAF5, CoAF5, BCAMF5,

BMnAMF5, and BCoAMF5 and (C) Comparative Chromatograms Obtained from UHPLC-DAD of Fractions CAF6, MnAF6, CoAF6, BCMF6, BMnMF6, and BCoMF6.

Table MS1. NMR Data (^{1}H and ^{13}C) in MeOD for the Substances: Pyrophen (1), Nigragilin (2), Penicillquei B (3), Aurasperone D (4), Fonsecinone B (5), and Fonsecinone A (6)

**1**		**2**		**3**		**4**		**5**		**6**	
**C**	**δ ^{1}H (ppm); *J* (Hz)**	**C**	**δ ^{1}H (ppm); *J* (Hz)**	**C**	**δ ^{1}H (ppm); *J* (Hz)**	**C**	**δ ^{1}H (ppm); *J* (Hz)**	**C**	**δ ^{1}H (ppm); *J* (Hz)**	**C**	**δ ^{1}H (ppm); *J* (Hz)**
2	---	2	6.45 (1H, d, 14,8)	2	---	2-Me	2.14 (3H, s)	2-Me	2.43 (3H, s)	2-Me	2.39 (3H, s)
3	5.55 (1H, d, 2.4)	3	7.20 (1H, d, 11.1 and 14,7)	3	5.56 (1H, d, 2.1)	3	6.08 (1H, s)	3	6.07 (1H, s)	3	6.27 (1H, s)
4	---	4	6.30 (1H, m)	4	---	9	7.01 (1H, s)	5-OH	14.60 (1H, s)	5-OH	12.94 (1H, brs)
4-OMe	3.82 (3H, s)	5	6.13 (1H, m)	5	6.01 (1H, d, 2.1)	10	7.18 (1H, s)	6-OMe	3.44 (3H, s)	6	6.99 (1H, s)
5	6.01 (1H, d, 2.4)	6	1.86 (3H, d, 4.0)	6	---	5-OH	---	8-OMe	3.84 (3H, s,)	7	6.90 (1H, s)
6	---	2′	4.51 (1H, brs)	7	4.93 (1H, dd, 8.3 e 7.1)	6-OMe	3.48 (3H, s)	9	6.99 (1H, s)	8-OMe	3.74 (3H, s)
								10	7.15 (1H, s)		
7	4.95 (1H, d, 8.5)	3′	2.79 (1H, dd, 4.0 and 12.0)<br>2.58 (1H, dd, 4.0 and 12.0)	8	3.15 (1H, dd, 8.3 e 7.1)<br>2.99 (1H, dd, 8.8 e 13.8)	8-OMe	3.81 (3H, s)	2′-Me	2.50 (3H, s)	10-Me	3.36 (3H, s)
7-COMe	1.92 (3H, s)	5′	2.98 (3H, m)	9	---	8-OH	---	3′	2,93 (2H, d, *J*= 5,7)	2′-Me	2.05 (3H, s)
8	3.08 (2H, m)	6′	3.99 (1H, m)<br>3.38 (1H, m)	10/14	7.19-7.30 (2H, m)	3′	7.28 (1H, s)	5′-OH	14.57 (s, 3H)	3′	5.94 (1H)
1′	---	7′	1.31 (3H, d, 6.8)	11/13	7.19-7.30 (2H, m)	7′	6.23 (1H, d, 1,9)	6′-OMe	4.02 (3H, s)	5′-OH	14.86 (1H, brs)
2′/6′	7.22 (2H, m)		2.41 (3H, s)	12	7.19-7.30 (2H, m)	9′	6.44 (1H, d, 1,9)	7′	6.38 (d, *J*= 1,7)	6′-OMe	4.04 (3H, s)
3′/5′	7.28 (2H, m)	7′	6,36 (d, 1H, J= 2,2 Hz)	4-OMe	3.82 (3H, s)	2′-Me	2.42 (3H, s)	8′-OMe	3.65 (3H, s)	7′	6.36 (1H d, 2,2)
4′	7.20 (1H, m)	8′	2.41 (3H, s)			5′-OH	---	9′	6.14 (d, *J*= 1,7)	8′-Me	3.55 (1H, s)
		9′	0.98 (d, 1H, 6.8)			8′-OMe	4.03 (3H, s)	--	--	9′	6.12 (1H, d, 2,2)
